# AP2/ERF and R2R3-MYB family transcription factors: potential associations between temperature stress and lipid metabolism in *Auxenochlorella protothecoides*

**DOI:** 10.1186/s13068-021-01881-6

**Published:** 2021-01-15

**Authors:** Guanlan Xing, Jinyu Li, Wenli Li, Sin Man Lam, Hongli Yuan, Guanghou Shui, Jinshui Yang

**Affiliations:** 1 State Key Laboratory of Agrobiotechnology, College of Biological Sciences, China Agricultural University, Beijing, 100193 China; 2 State Key Laboratory of Molecular Developmental Biology, Institute of Genetics and Developmental Biology, Chinese Academy of Sciences, Beijing, 100101 China; 3 University of Chinese Academy of Sciences, Beijing, 100049 China

**Keywords:** Transcription factors, Acyltransferase, Cold stress, Heat stress, Lipidomics, Triacylglycerol

## Abstract

**Background:**

Both APETALA2/Ethylene Responsive Factor (AP2/ERF) superfamily and R2R3-MYB family were from one of the largest diverse families of transcription factors (TFs) in plants, and played important roles in plant development and responses to various stresses. However, no systematic analysis of these TFs had been conducted in the green algae *A. protothecoides* heretofore. Temperature was a critical factor affecting growth and lipid metabolism of *A. protothecoides*. It also remained largely unknown whether these TFs would respond to temperature stress and be involved in controlling lipid metabolism process.

**Results:**

Hereby, a total of six AP2 TFs, six ERF TFs and six R2R3-MYB TFs were identified and their expression profiles were also analyzed under low-temperature (LT) and high-temperature (HT) stresses. Meanwhile, differential adjustments of lipid pathways were triggered, with enhanced triacylglycerol accumulation. A co-expression network was built between these 18 TFs and 32 lipid-metabolism-related genes, suggesting intrinsic associations between TFs and the regulatory mechanism of lipid metabolism.

**Conclusions:**

This study represented an important first step towards identifying functions and roles of AP2 superfamily and R2R3-MYB family in lipid adjustments and response to temperature stress. These findings would facilitate the biotechnological development in microalgae-based biofuel production and the better understanding of photosynthetic organisms’ adaptive mechanism to temperature stress.

## Background

AP2/ERF superfamily was a large family of plant transcription factors, containing one or two conserved AP2 DNA-binding domains (60–70 conserved amino acid sequences) [1]. The AP2/ERF superfamily was further classified into four families: AP2, ERF, DREB, related to ABI3 and VP1 (RAV) and Soloists [2, 3]. AP2/ERF transcription factors played important roles in many biological processes, including plant development, reproduction, biosynthesis of primary and secondary metabolites, as well as adaptation to biotic and abiotic stresses [1]. ERF family proteins, especially DREB (dehydration-responsive element binding) subfamily proteins, played important roles in abiotic stress. Overexpression of these proteins could improve plant stress resistance. MYB family was also a large group in plant TFs and widely distributed in plants [4]. MYB proteins were defined by a highly conserved DNA-binding domain, consisting of 1–4 imperfect repeats (R) of about 52 amino acids [5]. Based on the number of adjacent repeats, MYB proteins were classified into four types: 1R-MYB, R2R3-MYB, R1R2R3-MYB and 4R-MYB. R2R3-MYB TFs contained two repeats at the N-terminal, and were the most common among these MYB family TFs [6]. R2R3-MYB TFs regulated plant-specific biological processes and participated in plant growth and development, secondary metabolism and stress response [7]. AP2/ERF superfamily and R2R3-MYB family TFs had been identified and functionally characterized in many species of plants. However, it remained poorly known whether AP2/ERF and R2R3-MYB TFs were associated with the response to temperature stress and transcriptional regulation of lipid metabolism in green algae.

Microalgae oil has great potential and provides an alternative to vegetable oils and fossil fuels. However, due to its high production cost, the commercial application of microalgae oil remained unsuccessful [8, 9]. The construction of genetically engineered algal strains was expected to accelerate the commercialization of microalgae oil. However, due to the complexity of the microalgae oil synthesis pathway and the regulation mechanism, the actual regulation effects of one single gene or co-regulation effects of multi-genes on improving oil production varied among different algae. Courchesne et al. [10] proposed for the first time that genetic engineering of microalgae with high oil production could be constructed by modifying transcription factors. Hu et al. [11] found that there were 11 TFs that had potential transcriptional regulation effects on lipid metabolism in microalgae and these 11 TFs belonged to the AP2, ERF, MYB and bZIP family, respectively. Furthermore, it had been proved that NRR1 of SBP family, bZIP1 of bZIP family and ROC40 of MYB-related family could increase oil production in *Chlamydomonas reinhardtii* [12–14]. Li et al. [15] confirmed that NobZIP1 could increase algae’s oil production without affecting its normal growth. However, the role of AP2/ERF and MYB TF family in lipid regulation of microalgae was relatively rarely reported. In fact, WEINKLED1 (WRI1) of AP2 family in *Arabidopsis thaliana* had been confirmed to be able to bind to the conserved AW-box sequence in the upstream region of fatty acid synthesis gene [16]. Kang et al. [17] heterologously expressed AtWRI1 from *Arabidopsis thaliana* in *Nannochloropsis salina*, which increased lipid production. PHR1 of MYB family had also been reported to have regulated lipid remodeling and triglyceride accumulation in *Arabidopsis thaliana* during phosphorus starvation [18]. Therefore, the systematic analysis of AP2/ERF and MYB TF families and the relationship between them and lipid metabolism would facilitate exploration of the relatively new research field of genetic engineering of algal strain.

*Auxenochlorella protothecoides* is a strain of unicellular microalgae that belong to the class Trebouxiophyceae (Chlorophyta) [19]. With the advances in cultivation strategies, *A. protothecoides* was found to be able to grow under autotrophic, heterotrophic and mixotrophic conditions, and have responded well to stress treatment, such as nitrogen starvation, addition of NaCl and low or high temperatures. High biomass and high lipid productivity had been achieved in *A. protothecoides* under heterotrophic and mixotrophic conditions not only in shake flasks, but also in large-scale bioreactors [20–26]. Temperature is a crucial factor for microalgae production [27]. Our previous studies had revealed that low and high temperatures triggered the increases of lipid contents of *A. protothecoides* at the expense of cell growth, and eventually influenced biomass and lipid productivities in different ways [28, 29]. Besides, LT and HT stresses, respectively, triggered increases of 18% and 8% in the degree of unsaturation of fatty acids. However, the degree of unsaturation of membrane glycerolipids remained unclear in *A. protothecoides.* Though the glycerolipid compositions of *A. protothecoides* UTEX 2341 had been identified [30, 31], there was a large blank in the knowledge of changes of glycerolipid profiles and glycerolipid metabolism in *A. protothecoides* in responses to temperature stimuli.

Transcriptomic analysis showed that the expression of AP2/ERF superfamily and R2R3-MYB family TFs changed significantly under temperature stress. Therefore, in this study, the integrative genome and transcriptome-wide analysis of AP2/ERF and R2R3-MYB TFs in *A. protothecoides* were conducted to obtain their molecular characterization. Glycerolipid analyses of *A. protothecoides* UTEX 2341 were performed under low-, normal and high-temperature conditions based on liquid chromatography–multiple reaction monitoring mass spectrometry. The analyses of expression profiles of lipid metabolism-related genes aimed at an in-depth uncovering of the patterns of glycerolipid metabolism triggered by temperature stress. In addition, the regulatory network that controlled temperature-induced lipid adjustments was investigated. This study would facilitate the understanding of the mechanism underlying lipid adjustments of microalgae under environmental stress, the identifying of gene targets, and the construction of new algae strains with higher triacylglycerol (TAG) contents and temperature tolerance capacity by genetical engineering.

## Results

### Identification of *A. protothecoides* AP2/ERF and R2R3-MYB genes

Twelve AP2/ERF and 6 R2R3-MYB proteins were identified in *A. protothecoides* (Additional file 2: Table S1). There were two proteins with three-AP2 domains, four proteins with two AP2 domains and six proteins with a single AP2 domain. A new *R2R3-MYB* gene, *ApMYB6*, was first identified by the transcriptomic analysis and then was successfully cloned (Accession Number: MT863608). There were far fewer AP2/ERF and R2R3-MYB family proteins in microalgae than in higher plants. The number of exons varied from 2 to 8 in AP2/ERF genes (Additional file 1: Fig. S1). According to the genome annotation, there were gap regions in *ApAP2-2*, *ApAP2-3* and *ApAP2-5* genes, the structures of which needed to be further analyzed. For R2R3-MYB genes, the number of exons ranged from 2 to 10, demonstrating highly different exon–intron structures even in the same subfamily.

### Conserved amino acid residues in AP2 and R2R3-MYB domains

**Fig. 1:**
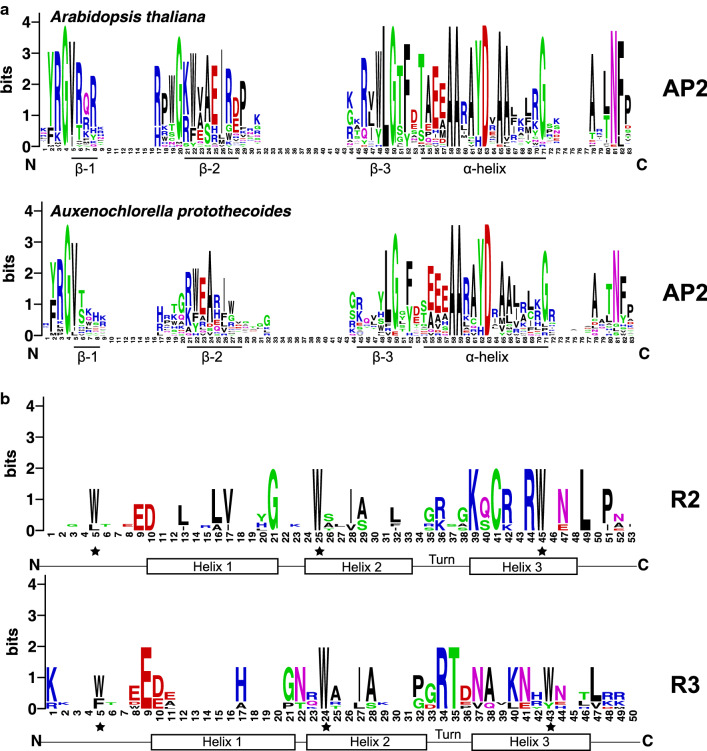
Sequence conservation of the conserved domain of AP2/ERF and R2R3-MYB proteins. **a** The sequence logos based on sequence alignments of AP2 domains of representative AP2/ERF proteins in Arabidopsis and all AP2 domains in *A. protothecoides*. The α-helix region and three β-sheets regions were shown. **b** The sequence logos based on sequence alignments of R2 and R3 MYB repeats of all R2R3-MYB proteins in *A. protothecoides*. The helix and helix-turn-helix domains that form each MYB repeat were labeled. The stars denote the typical, conserved tryptophan residues (W) in the MYB domain. The bit scores within each stack indicate the relative frequency of each amino acid at that position

To identify the conserved amino acid residues in AP2 domain, both sequences of AP2 domains from *A. protothecoides* and *Arabidopsis* AP2/ERF superfamily proteins were analyzed by multiple sequence alignments (Fig. 1a). The results showed that both *A. protothecoides* and *Arabidopsis thaliana* owned YRG and RAYD elements. However, there were low similarities among the sequences of three β chains of AP2 domains. The AAEIRD motif associated with target sequence recognition was only found in β-2 chain of the AP2 domains from ApERF3 and ApERF4. However, no DREB subfamily TFs were found in *A. protothecoides,* and the VAEIRE motif was found in none of the ERF family members either.

Sequence alignment among MYB domains from R2R3-MYB TFs of *A. protothecoides* (Fig. 1b) showed that the second and third tryptophans (W) in R2 domain were completely conserved, and the second tryptophan of R3 domain was completely conserved. However, the first tryptophan of R3 domain could be replaced by phenylalanine, and the third tryptophan could also be replaced by tyrosine. In addition to conserved tryptophan residues, other conserved amino acid residues in the MYB domains included G^21^, K^39^, C^41^, R^44^ and L^49^ in the R2 domain and E^9^, R^34^ and T^35^ in the R3 domain. Thereinto, K^39^ and C^41^ were located on the third α-helix of the R2 MYB domains.

### Phylogenetic analyses of AP2/ERF and R2R3-MYB family proteins

**Fig. 2:**
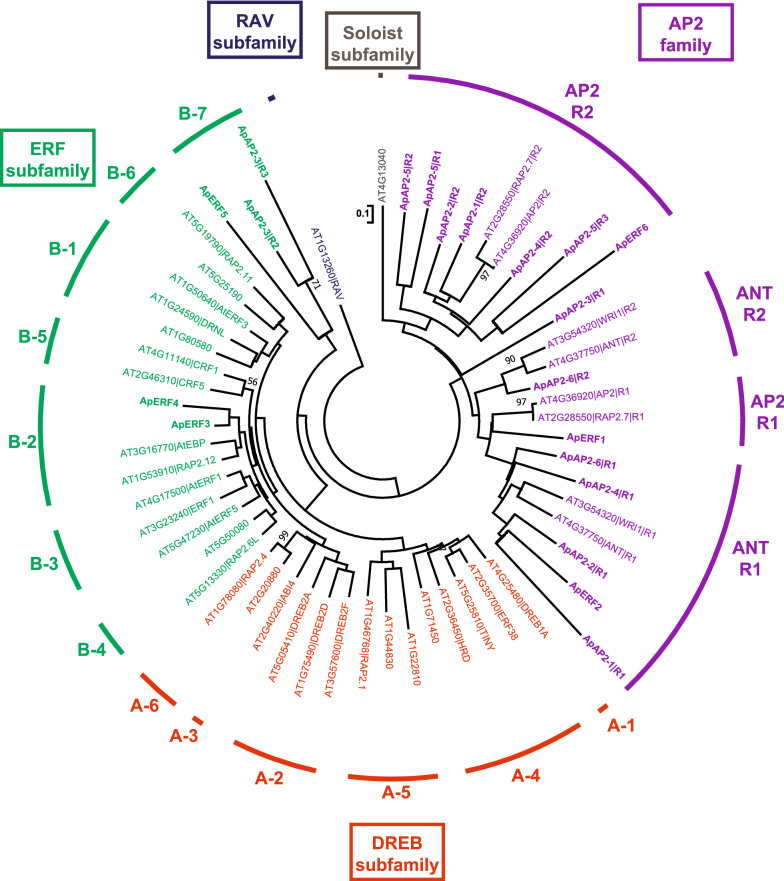
Phylogenetic relationships of AP2/ERF proteins from *A. protothecoides* (Ap) and *Arabidopsis thaliana* (At). The neighbor-joining (NJ) tree was constructed based on the amino acid sequences of the AP2/ERF domain using the JTT model and 1000 bootstrap samplings. Only support values with > 70% supports are indicated on the sides of important nodes. The classifications of subgroups in the AP2/ERF subfamilies are designated according to [2, 3]

To clarify the evolutionary relationships among and classifications of *A. protothecoides* AP2/ERF superfamily proteins, 20 *A. protothecoides* AP2 domains and 38 representative Arabidopsis AP2 domains were phylogenetically analyzed (Fig. 2). RAV and Soloist subfamilies were not found in *A. protothecoides*. ApAP2-1 R1, ApAP2-2 R1, ApAP2-4 R1, ApAP2-6 R1, ApERF1 and ApERF2 were classified into the ANT R1 group. ApAP2-6 R2 was classified into the ANT R2 group. ApERF1 belonged to the AP2 R1 group. ApAP2-1 R2, ApAP2-2 R2, ApAP2-4 R2, ApAP2-5 R1-3 and ApERF6 were classified into the AP2 R2 group. ApERF3 and ApERF4 belonged to the ERF family B2 group. ApERF5 and ApAP2-3 R2-3 were classified into a new subgroup B7 in the ERF subfamily. However, ApAP2-3 R1 belonged to none of the above branches, it was classified as a new group in the AP2 family*.*

**Fig. 3:**
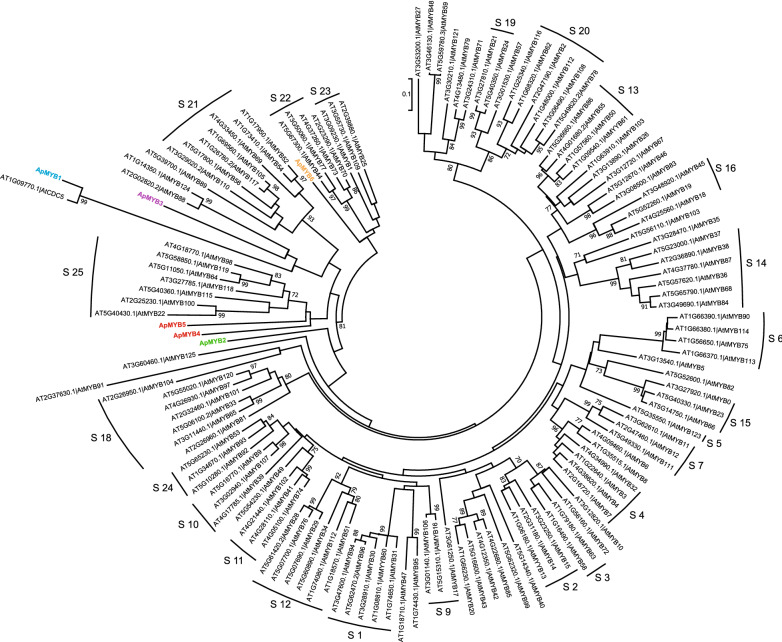
Phylogenetic relationships of MYB proteins from *A. protothecoides* (Ap) and *Arabidopsis thaliana* (At). The tree was inferred based on a complete protein sequence alignment of MYB proteins using the neighbor-joining (NJ) method, JTT model and 1000 bootstraps. The numbers at the branches > 70% are shown. The classifications of subgroups are marked as previously reported [6]

Phylogenetic analysis of 6 ApMYB proteins and 127 Arabidopsis R2R3-MYB proteins were carried out (Fig. 3). The results showed that ApMYB1 and AtCD5 shared high similarities and were clustered in the same cluster; ApMYB3, AtMYB88 and AtMYB124 were clustered in the same branch; ApMYB4 and ApMYB5 were close to the S25 subgroup of AtMYB proteins; ApMYB6, and S22 and S23 subgroups of AtMYB proteins were clustered together in the same branch; but ApMYB2 existed in different branches.

### Expression profiles of *ApAP2*, *ApERF* and *ApMYB* genes responded to temperature stress

**Fig. 4:**
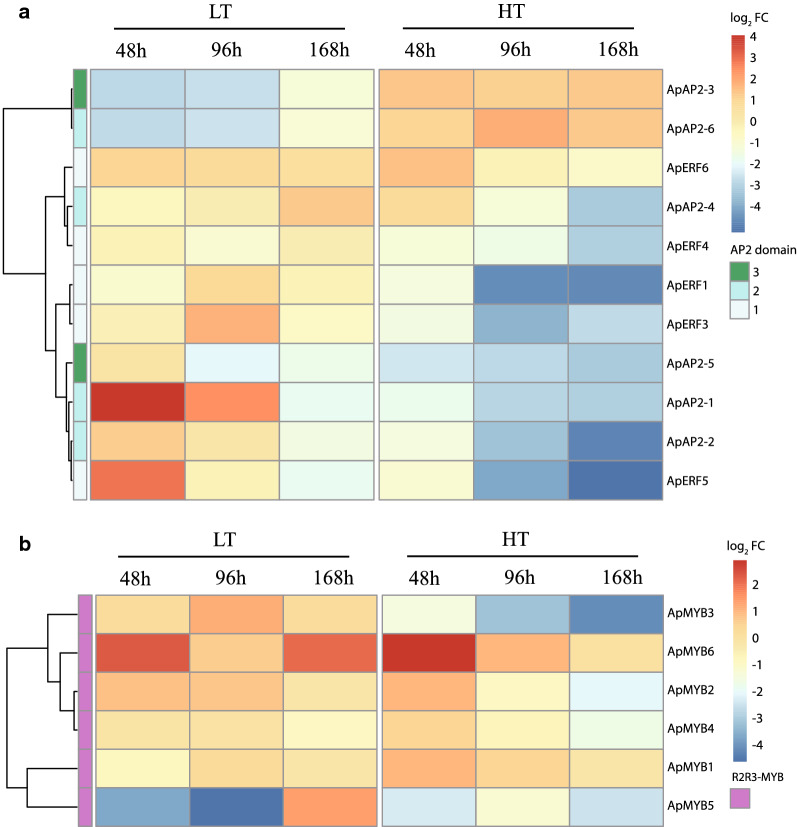
Clustergram representation of transcript level of AP2/ERF (**a**) and R2R3-MYB (**b**) proteins in response to low- and high-temperature stresses in *A. protothecoides* grown for 168 h. The hierarchical clustering by the Pearson rank correlation distance was conducted using the R-package ‘*Pheatmap*’

The changes in transcript abundance of all the TF genes during LT and HT stress were quantified using RT-qPCR analysis (Fig. 4 and Additional file 2: Table S2). All TF genes except for the expression of *ApERF1* were not detected by qPCR, which might have been caused by its low transcriptional abundance. *ApAP2-1*, *ApAP2-2*, *ApERF3* and *ApERF5* showed high expression level during the early stage under LT stress. In addition, their expression levels were down-regulated at 168 h, thus indicating that these four genes might have played a role in the adaptation to LT in the early stage. The expression of *ApAP2-4* was induced at 168 h, which indicated that *ApAP2-4* played an important role in the adaptation to LT stress in the later stage. It was noteworthy that *ApERF6* was continuously induced by LT. The expression of *ApAP2-3* and *ApAP2-6* was inhibited at LT, but up-regulated at HT, which showed that these two TFs were only induced by HT stress. Under HT stress, most of these TFs showed a downward trend in expression, including *ApAP2-1*, *ApAP2-2*, *ApAP2-5*, *ApERF1*, *ApERF3*, *ApERF4* and *ApERF5*.

Under LT stress, no obvious changes were detected in *ApMYB1*, *ApMYB2* and *ApMYB4* (|log_2_ FC| < 1), indicating that some members of R2R3-MYB family could not actively participate in the response to LT stress. Under HT stress, the transcription of *ApMYB1* was increased at 48 h (log_2_FC = 0.95), indicating that ApMYB1 might have responded well to temperature stress in the early stage. The expression of *ApMYB2-5* was down-regulated at 96 h and 168 h under HT stress. The similar variations of *ApMYB2* and *ApMYB4* genes indicated that they might have played similar roles under both LT and HT stresses.

### Glycerolipid profiles of *A. protothecoides* UTEX 2341

Enhanced lipid droplet accumulation was observed in BODIPY staining observation under LT and HT stresses (Additional file 1: Fig. S2). To uncover the glycerolipid changes of *A. protothecoides*, the glycerolipid profiles of cells grown after 96-h incubation under three temperature conditions were generated. The lipidomic analyses showed that 287 species of glycerolipids, including 3 classes of neutral lipids, 2 classes of galactolipids, 8 classes of phospholipids, 1 class of betaine lipids, were identified in *A. protothecoides* UTEX 2341 (Additional file 2: Tables S3 and S4). This was the first and most detailed report of glycerolipids in *A. protothecoides* under three different temperature conditions. However, due to the limitation of experimental conditions, sulfolipid sulfoquinovosyldiacylglycerol (SQDG) were not analyzed. The major phospholipids included phosphatidylcholine (PC), phosphatidylinositol (PI), phosphatidylethanolamine (PE) and phosphatidylglycerol (PG), accounting for 96 mol% (Additional file 1: Fig. S3). The other minor phospholipids were phosphatidylserine (PS), cardiolipin (CL), phosphatidic acid (PA) and lyso-phosphatidylethanolamine (LPE). PC accounted for more than 64 mol% of phospholipids and was the most abundant phospholipid, like in most eukaryotes. The relative abundance of galactolipids was far less than that of phospholipids. The content of monogalactosyldiacylglycerol (MGDG) was the highest among the canonical photosynthetic lipids [MGDG, digalactosyldiacylglycerol (DGDG) and PG]. Though diacylglyceryltrimethylhomo-serine (DGTS) had the lowest amount of all detected glycerolipids, the sum length of two acyl chain in DGTS was the longest in all lipid species, such as those of DGTS (44:3) and DGTS (44:4).

**Fig. 5:**
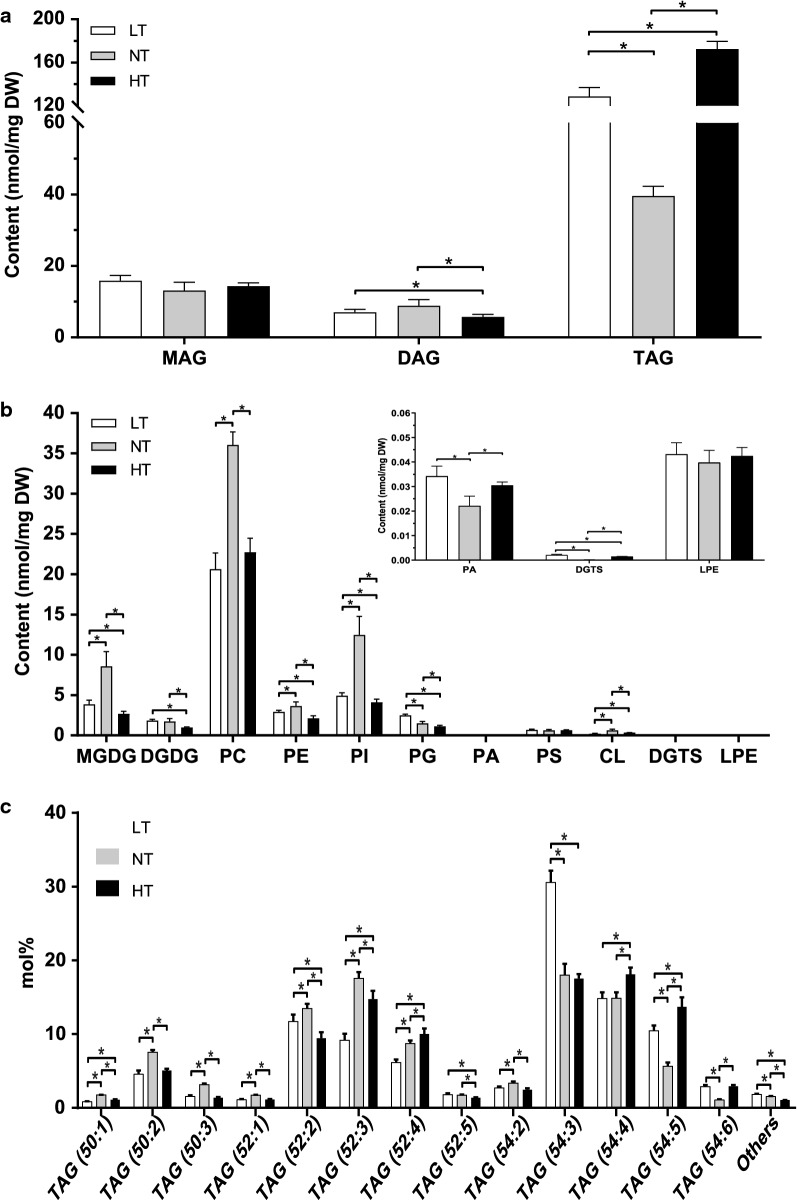
Analysis of glycerolipid species in *A. protothecoides* grown for 96 h under low-, normal- and high-temperature conditions. **a** The content of each class of neutral lipids. **b** The content of lipids assigned with each class of galactolipids, phospholipids and betaine lipids. **c** The relative abundance of each species of TAG. All values are mean ± SD of 4 biological repeats. *P* values indicate significant differences between two groups (Kruskal–Wallis test: *P < 0.05). *LT* low temperature, *NT* normal temperature, *HT* high temperature. The definitions of the abbreviations of lipids are shown in Additional file 2: Table S3

Principal coordination analysis showed obvious distinctions among glycerolipid profiles under different temperature conditions (Additional file 1: Fig. S4). Under LT stress, the contents of TAGs were increased by 2.3-fold and reached 68.0 mol% of the total glycerolipids (Fig. 5a and Additional file 1: Fig. S3). Under HT stress, the contents of TAGs were increased by 3.4-fold and reached 75.8 mol% of the total glycerolipids. These results showed that TAGs were major products of the temperature-induced lipid metabolic reprogramming. Six polyunsaturated TAGs, including TAG52:2, TAG52:3, TAG52:4, TAG54:3, TAG54:4 and TAG54:5 accounted for between 78.2 and 83.5 mol% of total TAGs (Fig. 5c). Besides, TAGs induced by LT and HT stresses showed preferences toward the biosynthesis of polyunsaturated species, such as TAG (54:5) and TAG (54:6).

The major membrane lipid classes decreased apparently in content under temperature stress, such as PC, PI, PE and MGDG (Fig. 5b and Additional file 1: Fig. S3). Under LT and HT stresses, the amounts of membrane glycerolipid species with total polar glycerolipids accounting for more than 5% decreased by different degrees. However, the amounts of the minor polar species increased, such as PS and DGTS. The contents of thylakoid membrane lipids, including DGDG and PG, decreased under HT stress. However, the content of PA significantly increased, and the proportion of DGDG in total membrane lipids increased from 3 to 5%.

Under LT stress, the double-bond index (DBI) of total membrane lipids increased from 2.46 to 3.41, and all the membrane glycerolipids classes increased apparently in DBI, from 10.5 to 97.6% (Additional file 1: Fig. S6). MGDG36:6 (18:3/18:3) accumulated to 2.53 nmol mg^−1^ DW and reached 65.3 mol% of MGDG (Additional file 1: Fig. S5). DGDG36:6 (18:3/18:3) accumulated to 1.32 nmol mg^−1^ DW and increased to 72.9 mol% of DGDG. Remarkable increases of 18:3 fatty acids in MGDG and DGDG were also observed under LT stress (Additional file 1: Fig. S7). Higher amounts of membrane lipids with C18:3, which contributed to fatty acid unsaturation degrees of cell membranes, well explained why *A. protothecoides* could adapt to cold stress.

Under HT stress, the DBI of the total lipids remained relatively stable (Additional file 1: Fig. S6). The DBI of PC, one of the major membrane lipid classes, only slightly increased by 2.7%. Interestingly, the DBI of galactolipids (MGDG and DGDG) significantly increased. Further analysis of the composition of galactolipids showed that the contents of C18:3 in galactolipids increased significantly (Additional file 1: Fig. S7). The above results showed that HT influenced the unsaturation of membrane lipids untypically, and the responses of thylakoid membrane lipids to HT were different from those of extraplastidial lipids. This untypical change might be one of the reasons why *A. protothecoides* are unable to adapt to higher temperatures above 32 ℃.

### RNA-seq analysis of glycerolipid metabolism

qPCR validation of the RNA-seq data was conducted in our previous study [28]. To further analyze glycerolipid metabolic adjustments at the transcript level, the pathways of glycerolipid metabolism in *A. protothecoides* UTEX 2341 were first constructed, and the differentially expressed genes affected by temperature stress extracted from our previous RNA-seq datasets were examined [28] (Additional file 2: Table S5). Genes with *q*-value < 0.05 were assigned as differentially expressed genes.

**Fig. 6:**
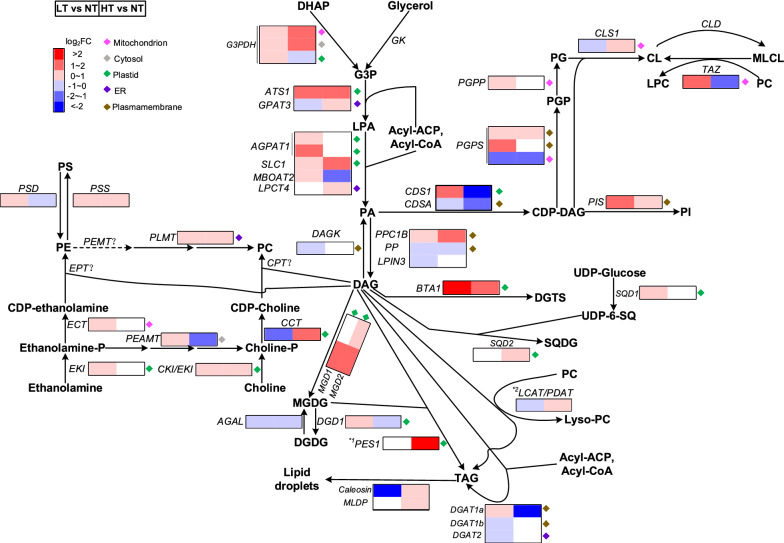
Genomic and transcriptomic features of glycerolipid biosynthesis in *A. protothecoides*. Heat map of differential expression of glycerolipid biosynthesis genes under low- and high-temperature stress conditions was constructed with HemI. The alterations of these genes at the transcript level were obtained according to our previous transcriptomic study [19]. End products are shown in bold. Genes encoding the enzymes are labeled in italic. Solid arrows indicate direct enzymatic steps, and dashed arrows represent multiple enzymatic steps. ^*1^: TAG biosynthesis mediated by PES1 is drawn here in the plastid. But so far, the evidence for existence of this reaction is still lacking in green algae. ^*2^: It remains unknown that whether the LCAT/PDAT is a TAG biosynthesis enzyme in *A. protothecoides*. The definitions of the abbreviations are shown in Additional file 2: Tables S3 and S5. *LT* low temperature, *NT* normal temperature, *HT* high temperature

Under LT stress, genes that involved in de novo TAG biosynthesis were up-regulated at the transcript level, including three *glycerol-3-phosphate dehydrogenase* genes (*G3PDH*; F751_1461, F751_3648 and F751_6745), one glycerol-3-phosphate acyltransferase gene (*ATS1*; F751_0154), four *lysophospholipid acyltransferase* genes (F751_2851, F751_2852, F751_3831 and F751_3591), one phosphatidate phosphatase gene (*PPC1B*; F751_1569) and one *diacylglycerol acyltransferase gene* (*DGAT1a*; F751_1386) (Fig. 6). Meanwhile, the down-regulation of the gene encoding diacylglycerol kinase (DAGK) at the transcript level was observed. Under HT stress, two *G3PDH* genes (F751_1461, F751_3648), two glycerol-3-phosphate acyltransferase genes (*ATS1* and *GPAT3*; F751_0154 and F751_5853), two lysophospholipid acyltransferase genes (*SLC1* and *LPCT4*; F751_3831 and F751_2508), and *PPC1B* were up-regulated at the transcript level. It is noteworthy that under HT stress, only two genes encoding acyltransferase-like proteins (*LCAT/PDAT* and *PES1*) were up-regulated by 1.9-fold and 8.2-fold at the transcript level. The induction of these two genes well explained higher TAG content under HT stress than under LT stress. The results demonstrated that these two genes were closely related to enhanced TAG accumulation in *A. protothecoides*. Putative TAG, DAG and MAG lipases were found in *A. protothecoides* (Additional file 1: Fig. S8). Under LT and HT stresses, the transcripts of most genes involved in TAG degradation were down-regulated, which reflected depressed TAG catabolism.

Consistent with metabolite analysis of betaine lipids (Fig. 5b), the transcript level of the gene encoding DGTS synthesis enzyme (*BTA1*) was elevated under LT and HT stress (4.7-fold and 3.7-fold, respectively) (Fig. 6). The results suggested that DGTS biosynthesis could be induced in *A. protothecoides* under temperature stress. Under LT stress, some genes involved in PG biosynthesis exhibited a synchronized increase at the transcript level, including one *phosphatidate cytidylyltransferase* gene (*CDS1*; F751_4726), two *CDP-DAG-glycerol-3-phosphate 3-phosphatidyltransferase* genes (*PGPS*; F751_0410 and F751_5216) and one *phosphatidyl glycerophosphatase* gene (*PGPP*; F751_3869), which kept up with the increase of PG. Under HT stress, some genes of PG biosynthesis exhibited the patterns of decreased expression, including two *phosphatidate cytidylyltransferase* genes (*CDS1* and *CDSA*; F751_4726 and F751_5891) and one *PGPS* (F751_0645).

### Transcriptional dynamics of lipid metabolism under low- and high-temperature stresses

**Fig. 7:**
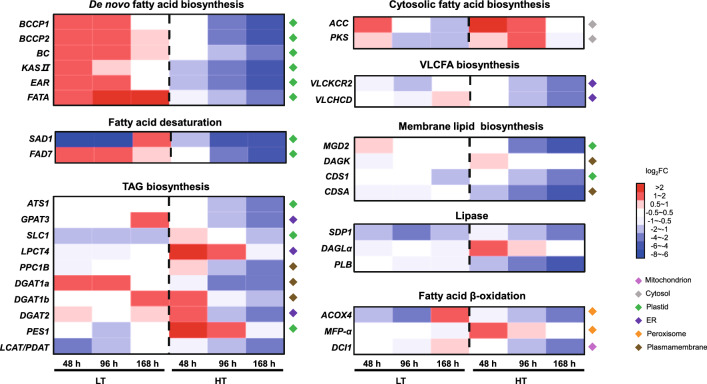
Transcriptional dynamics of 32 individual genes associated with lipid metabolism in response to low- and high-temperature stresses in *A. protothecoides* grown for 168 h. The transcript levels of *FAD7* in response to low-temperature stress and *ACC* in response to high-temperature stress were obtained according to our previous study [49]. The heatmap was generated using HemI. The definitions of the abbreviations are shown in Additional file 2: Table S7. *LT* low temperature, *NT* normal temperature, *HT* high temperature

Integrated analysis of lipid metabolite and transcriptomic profiling unveiled the important candidate genes that were associated with lipid adjustment to LT and HT stresses. To gain insight into the dynamic transcriptional changes of lipid metabolism under LT and HT stresses, transcripts for 32 candidate genes involved in lipid adjustment were tested by RT-qPCR (0 h, 48 h, 96 h and 168 h) (Fig. 7 and Additional file 2: Table S7).

Under LT stress, all six genes involved in de novo FA biosynthesis were up-regulated at 48 h and 96 h (Fig. 7). Thereinto, the gene encoding oleoyl-ACP thioesterase (*FATA*; F751_4313*)* remained highly expressed at 168 h. The enhanced expression of these genes indicated that LT stress continuously activated de novo FA biosynthesis. In de novo TAG biosynthesis, *GPAT3*, *DGAT1a* and *DGAT1b* were up-regulated and *SLC1* was down-regulated at the transcript level. At 48 h and 96 h, only *DGAT1a* in all of the three *DGAT* genes was up-regulated, which further indicated that *DGAT1a* might have played a role in early TAG accumulation and the early response to LT stress. The down-regulation of *PES1* and *LCAT/PDAT* at the transcript level indicated that TAG biosynthesis from the membrane lipid transformation could be inhibited. The decreased transcripts of *triacylglycerol lipase sugar dependent 1* (*SDP1*; F751_6987) and *acyl-CoA oxidase 4* (*ACOX4*; F751_5463) were also observed. In addition, the high expression of ω-3 fatty acid desaturase (*FAD7*; F751_6504) was closely related to the dramatic increase of ω-3 fatty acids at LT. The expression of *acyl-ACP desaturase 5* (*SAD1*; F751_6440) was dramatically changed, suggesting that *SAD1* could be closely involved in the algae’s adaptation to LT.

Under HT stress, the six genes involved in the de novo FA biosynthesis pathway were down-regulated (Fig. 7), but up-regulated *Acetyl-CoA carboxylase* (*ACC*; F751_4884) and polyketide synthase (*PKS*; F751_4316) were observed, suggesting that HT activated cytosolic FA biosynthesis. In de novo TAG biosynthesis, up-regulated genes included *LPCT4*, *DAGT1b* and *DGAT2*. The increase of *PES1* indicated activated TAG biosynthesis from membrane lipid transformation under HT stress. The down-regulation of *CDS1*, *CDSA* and *MGDG synthase* gene (*MGD2*; F751_2605) was also observed, suggesting the inhibited biosynthesis of phospholipids and glycolipids. Some genes involved in lipid desaturation and degradation were down-regulated, including *FAD7*, *SAD1*, *SDP1*, *ACOX4* and one *Δ*^*3,5*^*, Δ*^*2,4*^*-dienoyl-CoA isomerase* gene (*DCI1*; F751_6219).

### Establishment of lipid metabolism regulatory networks

**Fig. 8:**
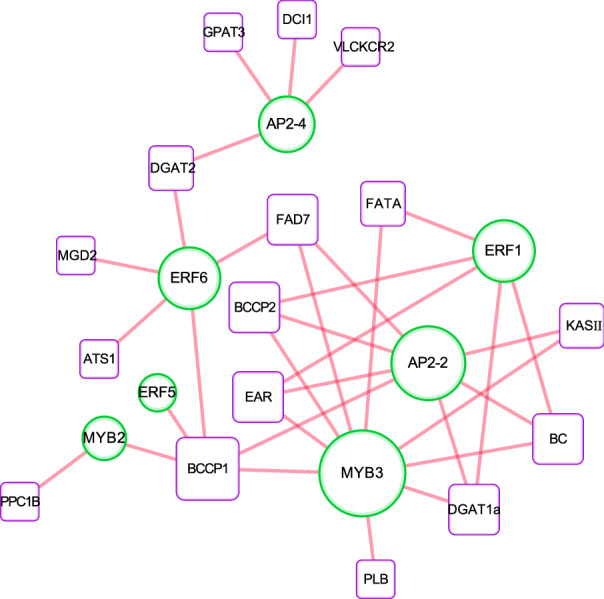
Cytoscape visualization of the lipid metabolism regulatory network in *A. protothecoides* under temperature stress. The co-expression network that included 17 TFs and 32 metabolism-related genes was constructed using the Pearson coefficient (*R* ≥ 0.9 and *P* < 0.005) as a cutoff threshold value. Green circles represent TF genes and purple squares indicate lipid metabolism-related genes. Red lines indicate positive significant correlations. The full names of the corresponding genes are shown in Additional file 2: Tables S2 and S7

According to the temporal changes of 17 genes encoding AP2/ERF and R2R3-MYB TFs and 32 genes involved in lipid metabolism during LT and HT stresses, the expression correlation was calculated based on the log_2_-transformed fold changes. The Pearson correlation coefficients (|*R*|> 0.9) and *P* < 0.05 were used as the criteria. No positive correlation was observed in the regulatory network, indicating that functions of these TFs were transcriptionally activated. The transcriptional networks consisted of 7 TFs that acted as hubs, connected with 16 putative downstream lipid-related genes (Fig. 8). *ApMYB3* and *ApAP2-2* had the two largest number of statistical interactions with downstream genes. Thereinto, *ApMYB3* were inferred to have regulated a total of 8 genes. The results suggested that they might have served important roles in lipid adjustment and TAG accumulation. *BCPP1* was the most commonly regulated lipid gene by putative regulators, including *ApAP2-2*, *ApERF5*, *ApERF6*, *ApMYB2* and *ApMYB3*.

## Discussion

Acyltransferases played a key role in the synthesis and accumulation of microalgae TAG [32]. It is well known that DGATs could catalyze the last acylation step of TAG biosynthesis, and are considered as a key enzyme that could greatly limit the inducing of TAG accumulation. In addition, heterotrophic cultivation was reported to have activated higher transcript abundance of one gene encoding DGAT and led to TAG accumulation in *A. protothecoides* [33]. In this study, data showed that the three DGATs in *A. protothecoides* varied in their responses to LT and HT stress. In *Arabidopsis thaliana*, DGAT1 was found to have contributed to freezing tolerance [34, 35]. This study showed that *ApDAGT1a* was a highly cold responsive gene, and its higher expression was closely related to TAG accumulation under LT stress. *ApDAGT1b* and *ApDGAT2* were early heat responsive genes, and could participate in the adaption to HT stress and early TAG accumulation. The close relationship between *ApDGATs* and temperature stress to some extent reflected their roles in the algae’s tolerance to abiotic stress, which had been neglected before. The phospholipid:diacylglycerol acyltransferase (PDAT) had been proven to have directly catalyzed the conversion of membrane lipids to TAG in *Chlamydomonas reinhardtii* [36]. The *Chlamydomonas* homologous PDAT protein was found in many microalgae [37]. Whether there were other proteins in microalgae that could directly catalyze the transformation of membrane lipids into TAG was still unclear. Except for the putative PDAT proteins, the homologs of *Arabidopsis* PES1 could all participate in the lipid turnover of *A. protothecoides*. The PES1 (AT1G54570) of *Arabidopsis* was known to possess diacylglycerol acyltransferase activities and could employ acyl-CoAs, acyl carrier proteins, and galactolipids as acyl donors to yield TAGs and had a high expression during senescence and under nitrogen deprivation [38]. By sequence alignment analysis, it was found that ApPES1 was 33.06% consistent with PES1 from *Arabidopsis thaliana*, and 30.65% consistent with PES2 from *Arabidopsis thaliana* in the protein sequences. Phylogenetic analysis showed that there were many PES homologous proteins in many unicellular species of green algae, including *Chlorella sorokiniana*, *Chlamydomonas reinhardtii* and *Ostreococcus tauri* (Additional file 1: Fig. S9). It was speculated that the theory that acyl groups from thylakoid galactolipids could directly transform to TAG mediated by PES1 might be universally applicable to green algae. It was important to note that the expression of *ApPES1* was highly heat responsive. The overexpression of the putative PES1 might contribute to augments in basal thermotolerance, similar to the role of PDAT in *Arabidopsis thaliana* [39].

With the rapid development of next-generation sequencing technology, AP2/ERF and R2R3-MYB TFs had been widely identified in a large number of plants, and their numbers varied in different plants. In addition, some professional plant TF databases had been built to provide study tools for researchers, such as PlantTFDB and PlnTFDB. Green algae had a smaller number of AP2/ERF and R2R3-MYB TFs, compared with Embryophyte [40, 41]. The vast expansion of these superfamily TFs was related to more than one genome-wide duplication event during the evolution of higher plants. It was observed that *A. protothecoides* harbored only 10 AP2/ERF family TFs and no DREB subfamily TFs. The results further supported the notion that AP2/ERF superfamily TFs could originate from the AP2 family TFs and ERF subfamily TFs was the older forms of ERF family than DREB subfamily. Some TFs contained only one-AP2 domain (ApERF1 and ApERF2), which showed higher similarity to the AP2 domains contained in double/three-AP2 proteins than the other ERF TFs’ AP2 domains. As mentioned in the literature review [1], a small number of one-AP2 proteins had been classified into AP2 family based on high similarities. However, plenty of studies agreed with the definition that the AP2 TFs contained only two AP2 domains. However, AP2 proteins containing more than two (3 and 7) AP2 domains were found in green algae [41], indicating that the traditional classification of AP2/ERF superfamily TFs needed to be revised, which would be of great help to the understanding of the evolution and functions of these TFs containing AP2 domains.

A total of five R2R3-MYB TFs were present in the *A. protothecoides* genome and one additional R2R3-MYB was found in the assembled transcriptome. Some R2R3-MYB TFs of *A. protothecoides* shared high levels of sequence similarity with Arabidopsis R2R3-MYB TFs and were clustered into the known Arabidopsis functional clades. This would provide reference values to the function prediction of R2R3-MYB proteins of *A. protothecoides*. Prior studies had noted that the subgroup 25 of Arabidopsis R2R3-MYB family TFs played roles in seed development and nutrient distribution and storage [42–44]. For example, *AtMYB96* regulated seed TAG accumulation by activating *DGAT1* and *PDAT1* [43]. *AtMYB118* elevated the expression level of *LEAFY COTYLEDON1* (*LEC1*), thereby promoting the expression of fatty acid biosynthetic genes [44, 45]. Hence, it could be hypothesized that *ApMYB4* and *ApMYB5* were involved in the lipid biosynthetic process. The subgroup 22 of Arabidopsis R2R3-MYB family TFs, including *AtMYB44*, *AtMYB73*, and *AtMYB77*, were associated with stress responses, and transiently activated by cold stress [46, 47]. Based on the results of rapid up-regulation of *ApMYB6* under LT and HT stress, it was speculated that *ApMYB6* were involved in the early cold and heat stress responses.

Extensive studies had shown that members of AP2/ERF superfamily and R2R3-MYB family TFs were associated with the regulation of lipid metabolism in higher plants. For instance, *WRINKLED1* (*WRI1*), one Arabidopsis AP2 family TF, could activate the expression of fatty acid metabolic genes-*BCCP2*, *KAS* and *ACP1* in the early stage of seed development, and played an important role in seed maturation and oil accumulation [16, 48, 49]. Compared with *AtMYB96* and *AtMYB118* mentioned above, *AtMYB30* could control very-long fatty acid biosynthesis [50], while *AtMYB89* could repress seed lipid accumulation [51]. Studies on *Jatropha curcas* had shown that a transcription factor of R2R3-MYB family TF, *JcMYB1*, could bind to the promoter sequence of *DGAT1* and activate the expression of targeted genes [52]. Yet the association between these two families and lipid metabolism in green algae remained largely unexplored. With the advancement of omics technologies and the application of omics-based bioinformatic analysis, AP2/ERF superfamily and R2R3-MYB family TFs that were associated with lipid biosynthesis regulation had been predicted in green algae *Chlamydomonas reinhardtii* [53], *Chromochloris zofingiensis* [54] and *Nannochloropsis* [11]. Besides, based on the yeast one-hybrid assay and expression correlation analysis in *Chromochloris zofingiensis*, *CzMYB1*, a R2R3 family TF was speculated to have controlled TAG biosynthesis by regulating *CzDGTT5* [55]. Potential regulatory network hubs were also identified, which controlled lipid metabolism in *A. protothecoides* by co-expression analysis. Despite the potential association between the two families and lipid metabolism, further studies were required to confirm the functions of these TFs in green algae.

## Conclusion

In this study, the genome and transcriptome-wide analyses of AP2/ERF and R2R3-MYB TFs were performed in *A. protothecoides*, and their molecular characterization and expression profiles under LT and HT stress were obtained. Lipidomic analyses showed that TAGs were major lipid products of *A. protothecoides*. Integrated analysis of lipidomic and transcriptomic profiling revealed that some lipid genes were closely related to temperature-induced lipid adjustment, especially enhanced TAG accumulation. The transcriptional dynamics analyses of important lipid metabolism-related candidate genes further revealed distinct dynamic patterns of lipid adjustment. Based on the integrated analyses of the expression profiling of the AP2/ERF and R2R3-MYB TFs and the important candidate genes, a potential regulatory network that could control the temperature-induced lipid adjustments in *A. protothecoides* was proposed.

## Methods

### Identification of AP2/ERF and R2R3-MYB TFs in *A. protothecoides*

Previously, a comprehensive transcriptomic analysis of *A. protothecoides* UTEX2341 under LT and HT stress was performed [28]. All unigenes were queried by iTAK program (http://itak.feilab.net/cgi-bin/itak/index.cgi) to identify and classify TFs of *A. protothecoides* [56]. Based on the combined results of the AP2 and MYB domain analyses by InterPro tool (http://www.ebi.ac.uk/interpro/), SMART tool (http://smart.embl-heidelberg.de/), and plant transcription factor database (PlantTFDB, http://planttfdb.gao-lab.org/index.php?sp=Apr), the candidates with less than one-AP2 domain or two MYB domains were excluded from further analysis.

### Sequence alignment and phylogenetic analysis

A total of 34 representative AP2/ERF and 127 R2R3-MYB protein sequences of *Arabidopsis thaliana* were obtained from TAIR (http://www.arabidopsis.org/). Multiple sequence alignments of AP2/ERF and MYB domains were performed with ClustalW in conjunction with MEGA 7.0 using default settings. The sequence logos for these conserved domains were produced from the multiple sequence alignment files and visualized by the WebLogo tool (http://weblogo.berkeley.edu/logo.cgi) with default parameters. Phylogenetic trees were constructed using the neighbor-joining (NJ) method by the MEGA7.0 program based on the JTT model, using a bootstrap value of 1000 with default parameters.

### Strains and culture

*Auxenochlorella protothecoides* UTEX 2341 originated from the Culture Collection of Algae at the University of Texas (UTEX, USA). The medium composition was the same as previously described [57], including 17.5 g L^−1^ glucose, 13 g L^−1^ casein, 0.1 g L^−1^ yeast extract, 2 g L^−1^ NH_4_Cl, 1 g L^−1^ KH_2_PO_4_, 2 g L^−1^ Na_2_HPO_4_, 0.5 g L^−1^ MgSO_4_·7H_2_O, 0.01 g L^−1^ FeSO_4_, 0.01 g L^−1^ CaCl_2_ and 1 mL L^−1^ micronutrient solution [3.58 g Al_2_(SO4)_3_·18H_2_O, 12.98 g MnCl_2_·4H_2_O, 1.83 g CuSO_4_·5H_2_O and 3.2 g ZnSO_4_·7H_2_O per liter of distilled water]. The inoculum cultures were incubated at 28 ℃ under 50 μmol m^−2^ s^−1^ PAR in the dark/light cycles of 12-h Light/12-h Dark on an orbital shaker at 140 rpm for 48 h to reach the midlog phase (×10^8^ cells/mL) as of seeds. Then, they were inoculated at the ratio of 10% (v/v) into a 500-mL Erlenmeyer flask with 200 mL medium with orbital shaking at 140 rpm under different temperature conditions (10 ℃/28 ℃/32 ℃). Cells were cultivated without light illumination and any external supply of CO_2_. The experiments were repeated at least three times.

### Lipid extraction, LC–MS analyses, RNA extraction and RT-qPCR

Cells were collected at 96 h in 10-mL tubes, rapidly harvested by centrifugation, frozen in liquid nitrogen and stored at − 80 °C until lipid extraction. The lipid inactivation and extraction steps were performed twice and lipid extracts were pooled and dried. All LC–MS analyses were carried out on an Exion UPLC coupled to QTRAP 6500 Plus (Sciex, Framingham, MA). Further details of lipid extraction and LC–MS analyses were described in the Additional file 3: Additional methods. Total RNA extraction, cDNA synthesis and RT-qPCR were carried out as previously described [57]. Detailed protocols were shown in the Additional file 3: Additional methods.

### Statistical analysis

Data statistical analyses were performed using the SPSS 21 software. Data were presented as mean values ± SDs. For the statistical analyses of lipidomic data, non-parametric comparisons were performed by applying Kruskal–Wallis test (*P* < 0.05).

## Supplementary Information

**Additional file 1: Figure S1.** The exon–intron features of all the *AP2/ERF* and *R2R3-MYB* genes in *A. protothecoides*. Yellow boxes represent the exons. **Figure S2.** Confocal laser microscopy of *A. protothecoides* grown for 168 h under low, normal and high temperature conditions. **Figure S3.** Analysis of glycerolipid species in *A. protothecoides* grown at low, normal and high temperatures after 96-h cultivation. **Figure S4.** Principal coordinate analysis plot of the lipidomic data from *A. protothecoides* under three different temperature treatments. **Figure S5.** Changes in the content of the molecular species of membrane lipids in *A. protothecoides* grown for 96 h under low and high temperatures stress. **Figure S6.** The alterations of unsaturation levels of glycerolipids in *A. protothecoides* grown for 96 h under low and high temperature stress. **Figure S7.** Fatty acid composition changes of MGDG (A) and DGDG (B) in *A. protothecoides* grown for 96 h under low and high temperature stress. **Figure S8.** Heat map of differential expression of genes encoding lipases and genes involved in lipid transport under low and high temperature stress. **Figure S9.** Phylogenetic relationships of lipid acyltransferases from *A. protothecoides*, other green algae and plant.

**Additional file 2: Table S1.** The RNA-seq data for the putative AP2/ERF and R2R3-MYB transcriptional factors of *A. protothecoides*. **Table S2.** The relative transcript levels of AP2/ERF and R2R3-MYB genes in response to low and high temperature stress in *A. protothecoides* grown for 168 h. **Table S3.** The detailed information of lipid species identified in this study. **Table S4.** The contents of the glycerolipid molecular species in *A. protothecoides* grown for 96 h under low, normal and high temperature conditions. **Table S5.** The RNA-seq data for the putative genes in glycerolipid metabolism of *A. protothecoides* grown for 96 h under low and high temperature stress. **Table S6.** Subcellular localization predictions for candidate proteins involved in glycerolipid metabolism in *A. protothecoides*. **Table S7.** The relative transcript levels of 32 individual genes associated with lipid metabolism in response to low and high temperature stress in *A. protothecoides* grown for 168 h. **Table S8.** Primers sequences used for RT-qPCR analysis.

**Additional file 3.** Additional methods.

## Data Availability

All data used and/or analyzed during the current study were included in this article and the Additional files 1, 2.

## Abbreviations

ACC: Acetyl-CoA carboxylase
ACOX: Acyl-CoA oxidase
AP2: APETALA2
BL: Betaine lipid
CDS: Phosphatidate cytidylyltransferase
CL: Cardiolipin
DAG: Diacylglycerol
DAGK: Diacylglycerol kinase
DBI: Double-bond index
DCI1: Δ^3:5^:Δ^2:4^-Dienoyl-CoA isomerase
DGAT: Diacylglycerol acyltransferase
DGDG: Digalactosyldiacylglycerol
DGTS: Diacylglyceryl-*N*:*N*:*N*-trimethylhomoserine
DREB: Dehydration-responsive element binding
ERF: Ethylene Responsive Factor
FA: Fatty acid
FAD7: ω-3 Fatty acid desaturase
FATA: Oleoyl-ACP thioesterase
G3PDH: Glycerol-3-phosphate dehydrogenase
HT: High temperature
LPE: Lyso-phosphatidylethanolamine
LT: Low temperature
MAG: Monoacylglycerol
MGD: Monogalactosyldiacylglycerol synthase
MGDG: Monogalactosyldiacylglycerol
NT: Normal temperature
PA: Phosphatidic acid
PC: Phosphatidylcholine
PDAT: Phospholipid:diacylglycerol acyltransferase
PE: Phosphatidylethanolamine
PES1: Acyltransferase-like protein
PG: Phosphatidylglycerol
PGPP: Phosphatidylglycerophosphatase
PGPS: CDP-DAG-glycerol-3-phosphate 3-phosphatidyltransferase
PI: Phosphatidylinositol
PKS: Polyketide synthase
PS: Phosphatidylserine
R: Repeat
RAV: DREB: related to ABI3 and VP1
SAD: Acyl-ACP desaturase 5
SDP1: Triacylglycerol lipase sugar dependent 1
TAG: Triacylglycerol
TF: Transcriptional factor
