# Supplementary material for: AP2/ERF and R2R3-MYB family transcription factors: potential associations between temperature stress and lipid metabolism in Auxenochlorella protothecoides
Source: Biotechnol Biofuels. 2021 Jan 15;14:22. doi: 10.1186/s13068-021-01881-6 (PMC7811268; doi:10.1186/s13068-021-01881-6)
Supplement: Supplementary file 1 — Additional file 1: Figure S1. The exon–intron features of all the AP2/ERF and R2R3-MYB genes in A. protothecoides. Yellow boxes represent the exons. Figure S2. Confocal laser microscopy of A. protothecoides grown for 168 h under low, normal and high temperature conditions. Figure S3. Analysis of glycerolipid species in A. protothecoides grown at low, normal and high temperatures after 96-h cultivation. Figure S4. Principal coordinate analysis plot of the lipidomic data from A. protothecoides under three different temperature treatments. Figure S5. Changes in the content of the molecular species of membrane lipids in A. protothecoides grown for 96 h under low and high temperatures stress. Figure S6. The alterations of unsaturation levels of glycerolipids in A. protothecoides grown for 96 h under low and high temperature stress. Figure S7. Fatty acid composition changes of MGDG (A) and DGDG (B) in A. protothecoides grown for 96 h under low and high temperature stress. Figure S8. Heat map of differential expression of genes encoding lipases and genes involved in lipid transport under low and high temperature stress. Figure S9. Phylogenetic relationships of lipid acyltransferases from A. protothecoides, other green algae and plant. [file 13068_2021_1881_MOESM1_ESM.docx]

**
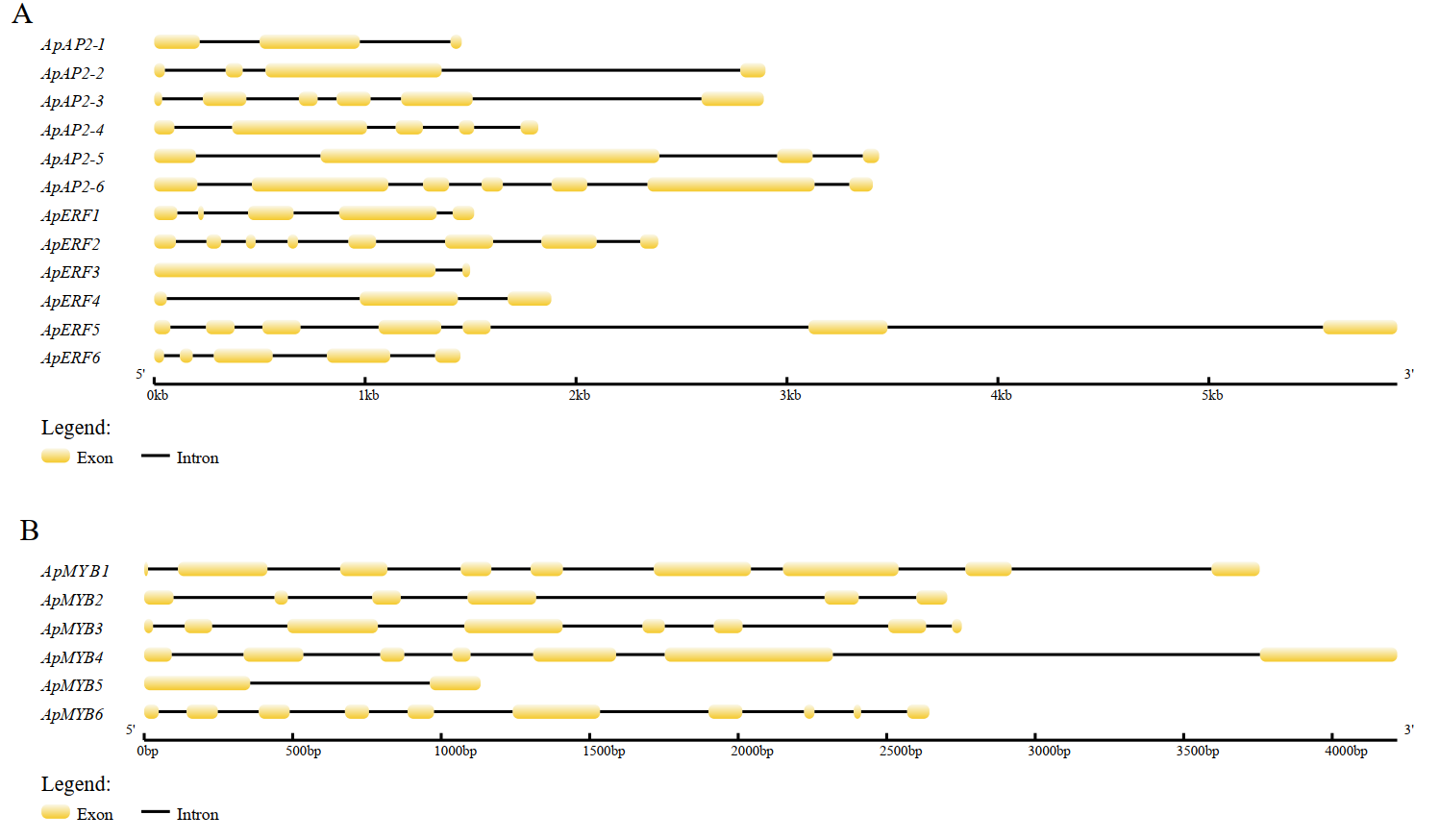
Fig. S1.** The exon–intron features of all *AP2/ERF* and *R2R3-MYB* genes in *A. protothecoides*. Yellow boxes represent the exons. Black lines indicate the introns. The horizontal axis (kb) indicate the lengths of genes.

**
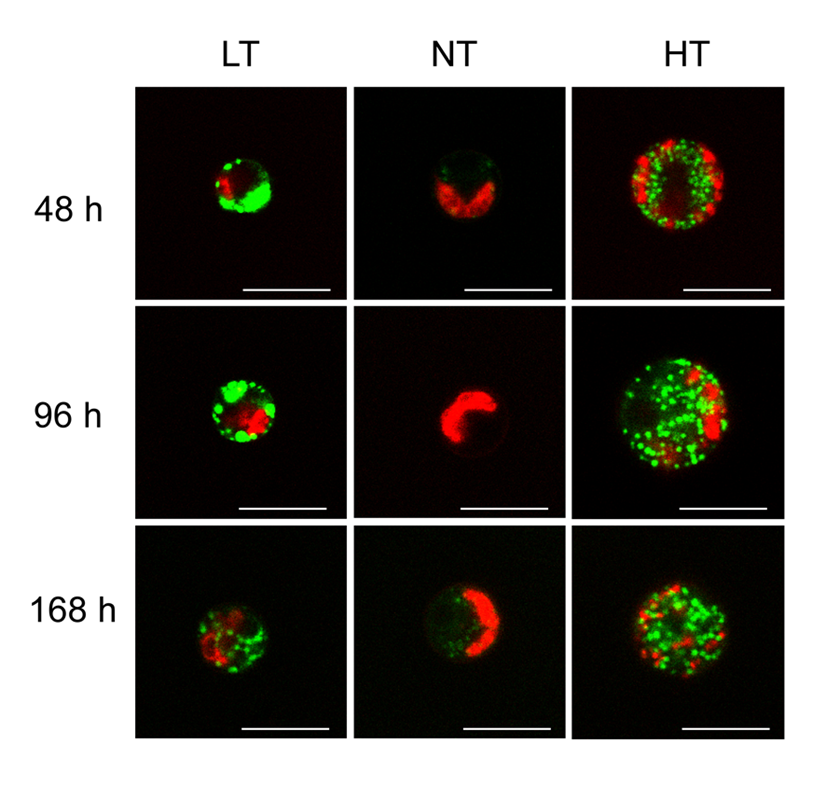
Fig. S2.** Confocal laser microscopy of *A. protothecoides* grown for 168 hours under low, normal and high temperature conditions. Cells were stained with BODIPY 505/515 to visualize lipid bodies (green color). Chloroplasts can be visualized by the chlorophyll autofluorescence (red color). Bars, 10 μm. LT, low temperature; NT, normal temperature; HT, high temperature.


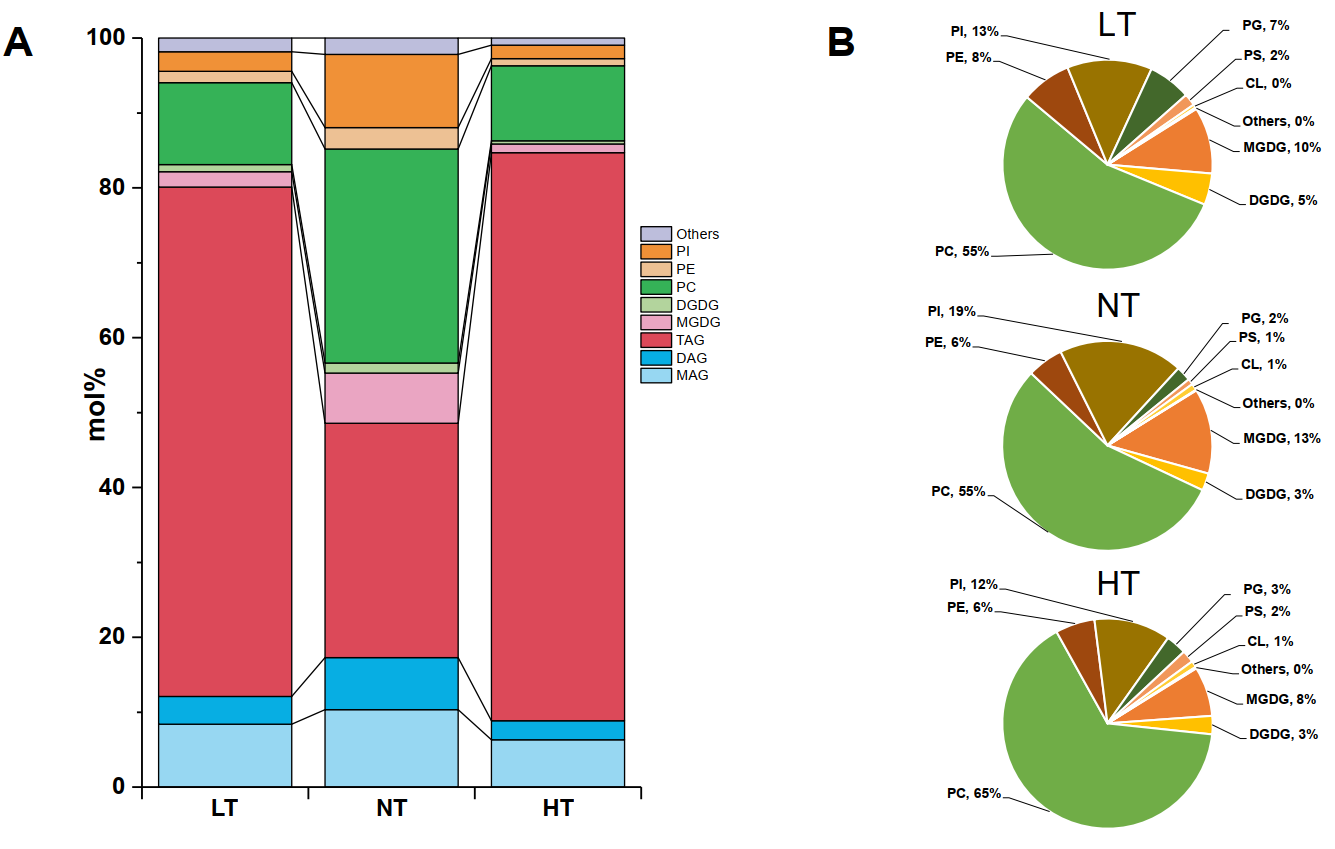


**Fig. S3.** Analysis of glycerolipid species in *A. protothecoides* grown at low, normal and high temperatures after 96-h cultivation. (A) Mol% of lipids assigned with the major lipid classes under three different temperatures conditions. (B) Molecular species of different lipid species of galactolipids, phospholipids and betaine lipids. All values are mean ± SD (*n* = 4). *P*-values indicate significant differences between two groups (Kruskal-Wallis test: *, *P* < 0.05). LT, low temperature; NT, normal temperature; HT, high temperature. The definitions of the abbreviations of each glycerolipid class are shown in Supplementary table S3.

**Fig. S4.** Principal coordinate analysis (PCoA) plot of the lipidomic data from *A. protothecoides* under three different temperature treatments. LT, low temperature; NT, normal temperature; HT, high temperature.
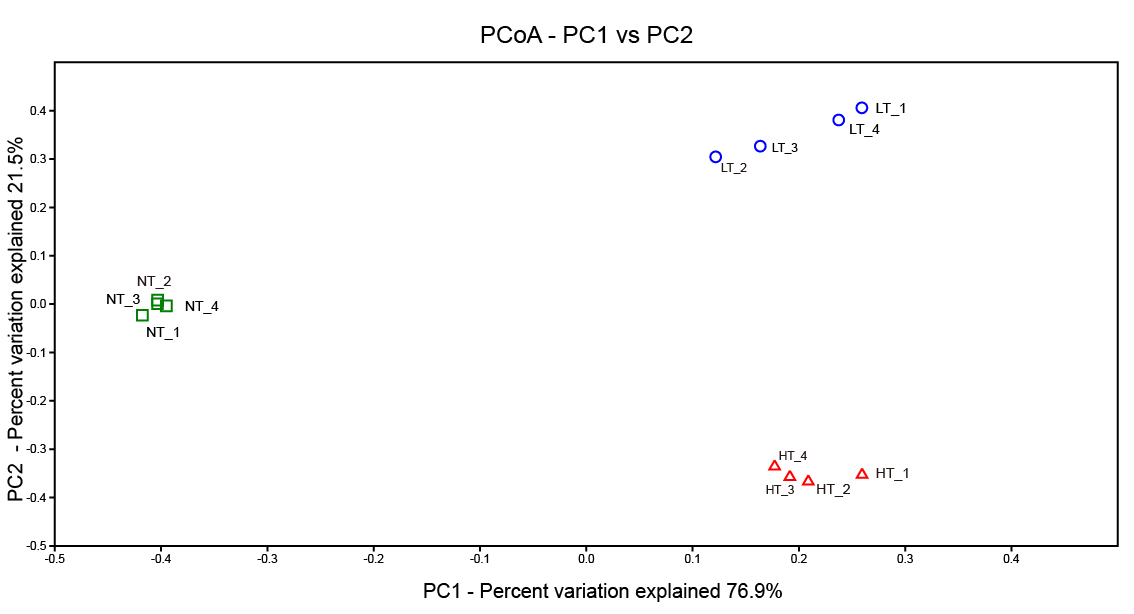


**
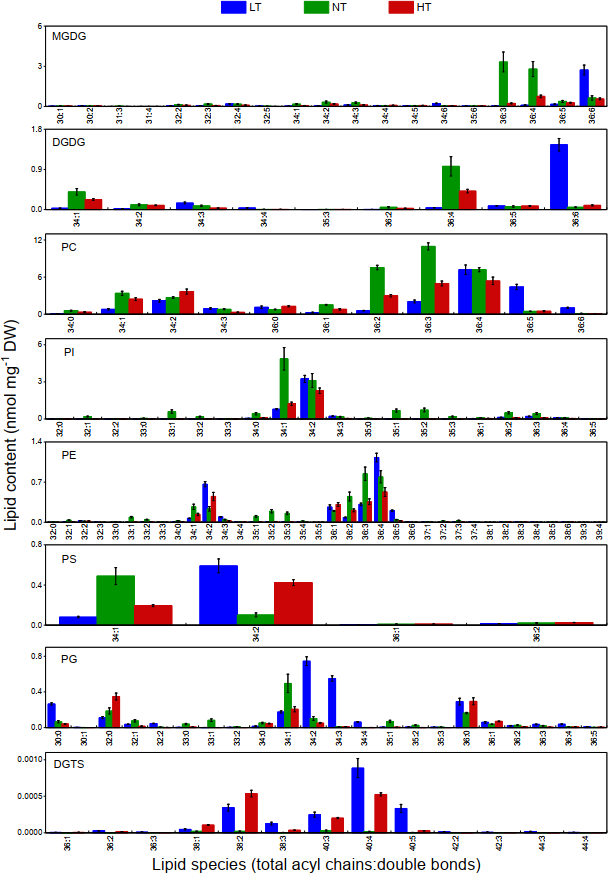
Fig. S5.** Changes in the content of the molecular species of membrane lipids in *A. protothecoides* grown for 96 hours under low and high temperatures stress. All values are mean ± SD (*n* = 4). Asterisks represent statistically significant differences using Kruskal-Wallis test: P < 0.05. LT, low temperature; NT, normal temperature; HT, high temperature. The definitions of the abbreviations of each glycerolipid class are shown in Supplementary table S3.


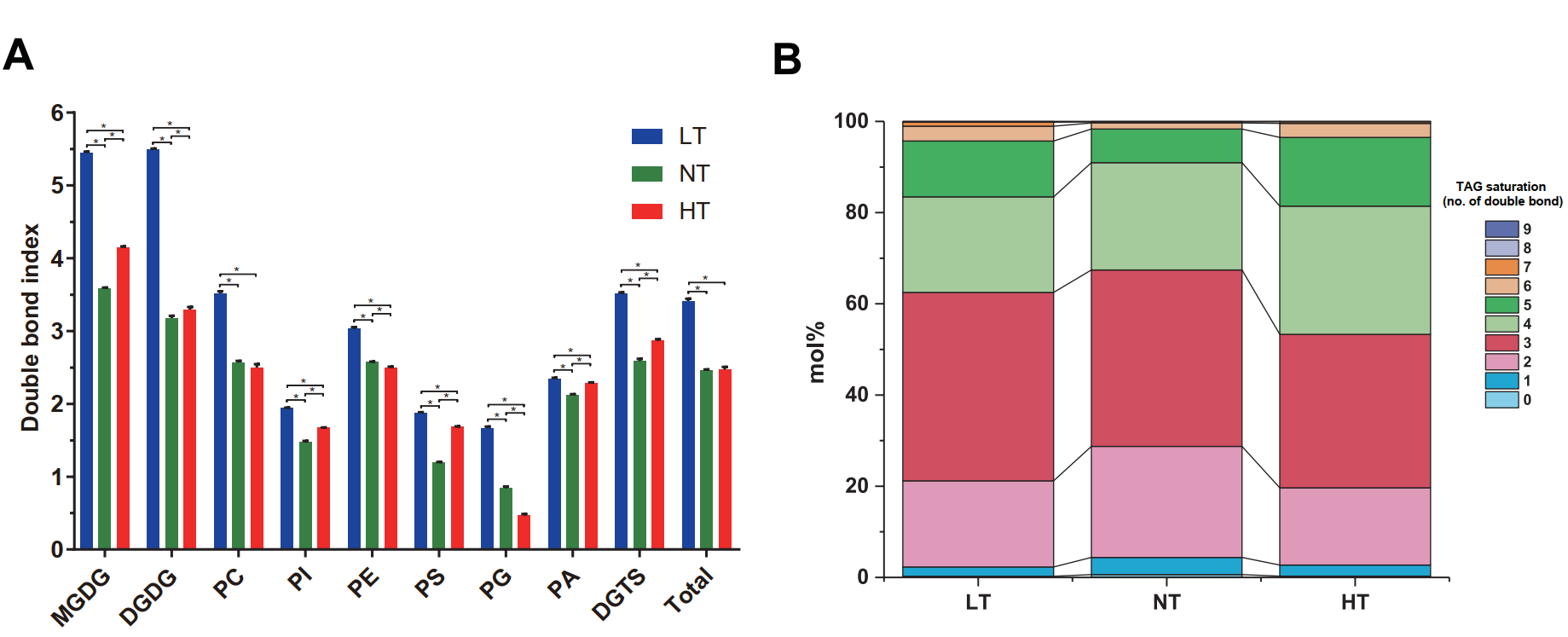


**Fig. S6.** The alterations of unsaturation levels of glycerolipids in *A. protothecoides* grown for 96 hours under low and high temperature stress. (A) Double bond index (DBI) of membrane lipids. DBI=$\sum[(N\times mol\% lipid)/100]$, N represents the total number of double bonds in the two fatty acid chains of each glycerolipid molecule. The definitions of the abbreviations of each glycerolipid class are shown in Supplementary table S3. (B) The relative abundance of TAGs with various unsaturation levels. The numbers (0-9) represent the sum of double bonds in TAGs. Values are means ± SD (*n* = 4). *P*-values indicate significant differences between two groups (Kruskal-Wallis test: *, *P* < 0.05).

**
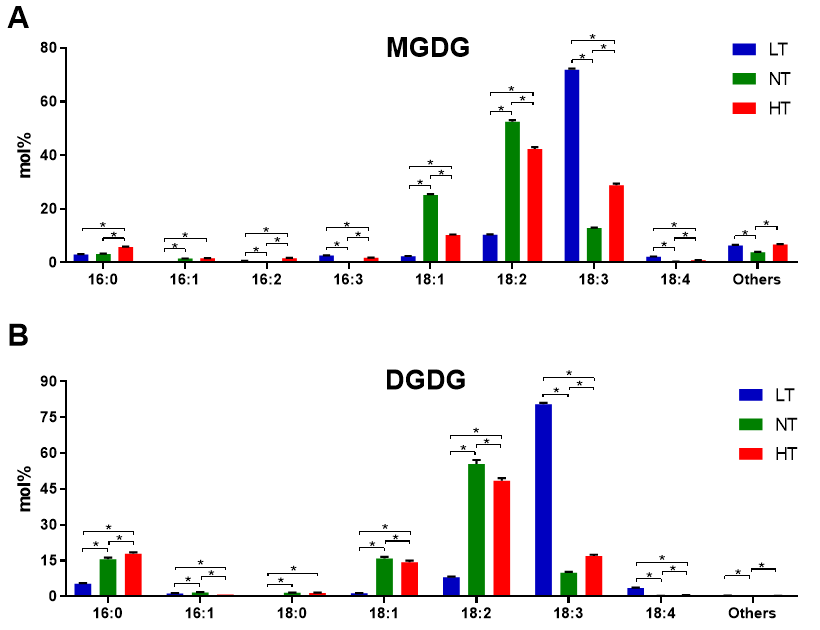
Fig. S7.** Fatty acid composition changes of MGDG (A) and DGDG (B) in *A. protothecoides* grown for 96 hours under low and high temperature stress. All values are mean ± SD (*n* = 4). Asterisks represent statistically significant differences using Kruskal-Wallis test: *P* < 0.05. MGDG, monogalactosyldiacylglycerol; DGDG, digalactosyldiacylglycerol.

**
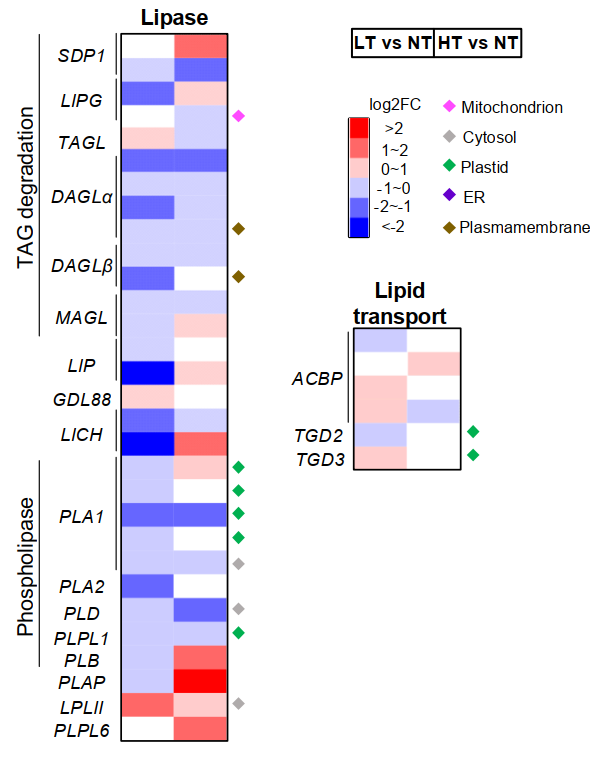
Fig. S8.** Heat map of differential expression of genes encoding lipases and genes involved in lipid transport under low and high temperature stress. The alterations of these genes at the transcript level were done according to our previous transcriptomic study (Xing *et al.*, 2018). The heat map was constructed with HemI. LT: low temperature; NT: normal temperature, HT: high temperature. The definitions of the abbreviations of each glycerolipid class are shown in Supplementary table S3.

**
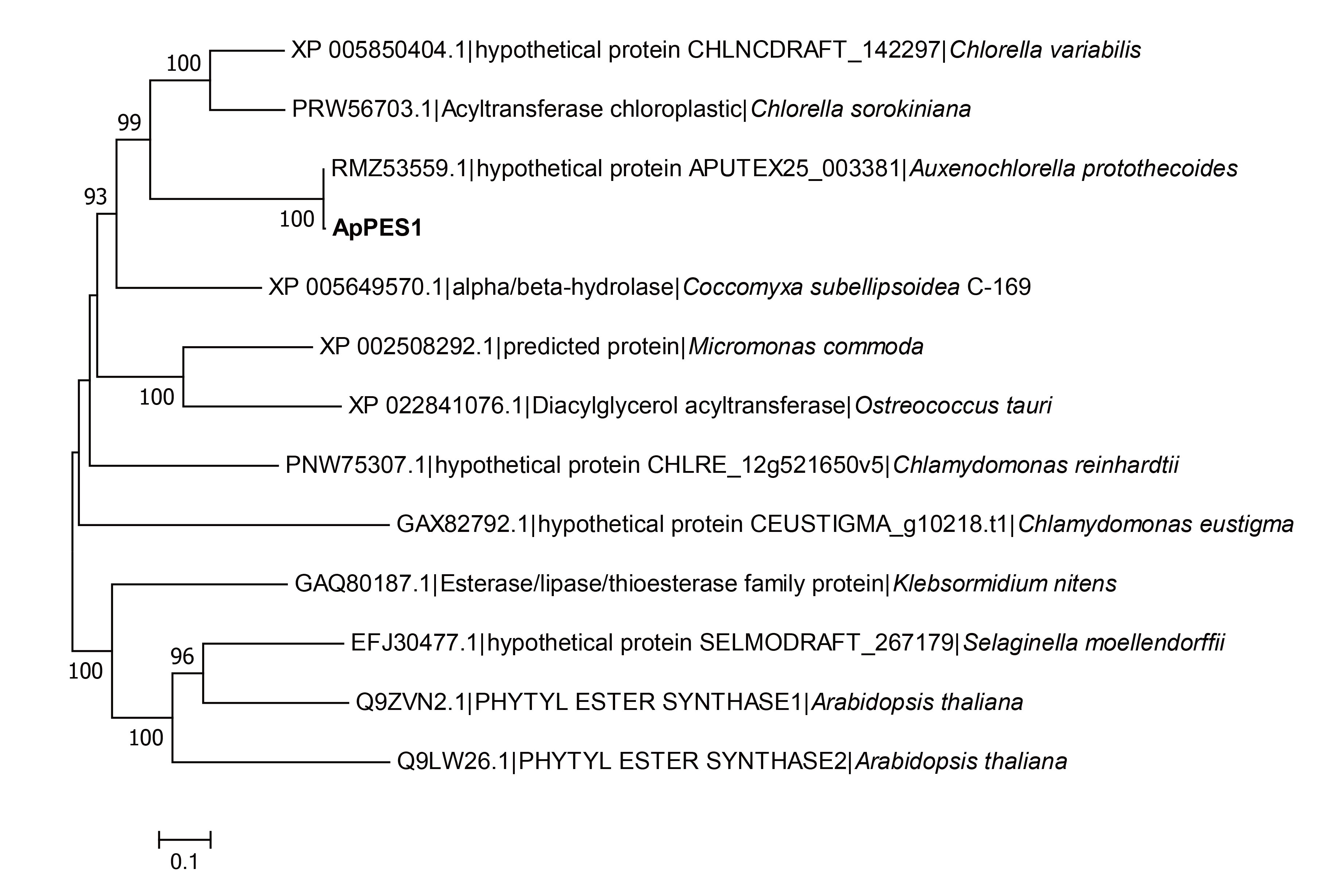
Fig. S9.** Phylogenetic relationships of lipid acyltransferases from *A. protothecoides*, other green algae and plant. The neighbor-joining (NJ) tree was constructed based on the Poisson model and 1000 bootstrap samplings. Only support values with >70% supports are indicated on the sides of important nodes.
