# Supplementary material for: AP2/ERF and R2R3-MYB family transcription factors: potential associations between temperature stress and lipid metabolism in Auxenochlorella protothecoides
Source: Biotechnol Biofuels. 2021 Jan 15;14:22. doi: 10.1186/s13068-021-01881-6 (PMC7811268; doi:10.1186/s13068-021-01881-6)
Supplement: Supplementary file 3 — Additional file 3. Additional methods. [file 13068_2021_1881_MOESM3_ESM.docx]

**Additional Methods**

**Gene structure analysis**

The genomic and coding DNA sequences of the identified AP2/ERF and R2R2-MYBTFs were retrieved from the genome database of NCBI (DDBJ/EMBL/GenBank accession: APJO00000000) [[1](#_ENREF_1)]. The exon–intron organizations were constructed and visualized by Gene Structure Display Server (GSDS) 2.0 software (http://gsds.cbi.pku.edu.cn/).

**Neutral lipid staining**

According to the previously described method [[2](#_ENREF_2)], microalgae cells were labeled with BODIPY^®^ 505/515 (Invitrogen) for 10 min at room temperature in the dark, using 1 μL of stain (25 μg mL^-1^, 2% DMSO [v/v] in water) per 100 μL of sample. In order to remove excess fluorescent dyes, cells were then washed with PBS buffer three times. The stained cells were observed by using Leica SP8 fluorescence microscopy.

**RNA extraction and RT-qPCR**

Total RNA was extracted from cells grown to the midlog phase and exposed to low and high temperature stress (10℃ and 32℃) for a certain number of hours (48, 96 and 168 hours), using Eastep^®^ Super Total RNA Extraction Kit (Promega, Shanghai, China). Reverse transcription reaction was performed on 1 μg of total RNA using GoScript™ Reverse Transcription Mix, Oligo(dT) (Promega, USA). qPCR reactions were prepared with 2× RealStar Green Power Mixture (Genstar, Beijing, China) and performed in the ABI Q6 (Applied Biosystems, USA) using the conditions: 95 °C for 10 min, 40 cycles at 95 °C for 15 s, and 60 ℃ for 1 min. The melt-curve analysis was conducted to check primer specificity. Ct values were calculated based on triplicate technical experiments performed on three biological replicates. The *RPL32* gene is used as an internal reference gene for normalization. Relative fold changes of selected genes were calculated based on the 2^-ΔΔCT^ method. Primer pairs used were shown in Supplementary Table S8.

**Lipid extraction**

After freeze-drying, the cell materials were directly weighed and inactivated with hot isopropanol, using a modified protocol as previously described [[3](#_ENREF_3)]. Following the inactivation, extraction solvent containing chloroform: methanol: 300 mM ammonium acetate (30: 41.5: 3.5) (v/v/v) was added to the samples, which were then incubated at room temperature for 24 h at 150 rpm. At the end of the incubation, samples were centrifuged and clear supernatant was transferred to fresh tubes. The lipid and extraction steps were repeated once and lipid extracts from both rounds of extraction were pooled and dried in a SpeedVac (GeneVac, Suffolk). Lipid extracts were stored at -80℃ until LC-MS analyses. Lipid extracts were stored at -80℃ until LC-MS analyses.

**UPLC-MS analysis**

For normal phase (NP) LC-MS, polar lipid analysis was conducted as previously described [[4](#_ENREF_4), [5](#_ENREF_5)]. Briefly, individual classes of polar lipids were separated by NP-HPLC with the use of a Phenomenex Luna 3μm-silica column (internal diameter 150 × 2.0 mm) under the following conditions: mobile phase A (chloroform: methanol: ammonium hydroxide, 89.5: 10: 0.5) and mobile phase B (chloroform: methanol: ammonium hydroxide: water, 55: 39: 0.5: 5.5). The gradient began with 95% mobile phase A for 5 min, followed by linear reduction to 60% mobile phase A over 7 min. The gradient was held for 4 min, and mobile phase A was then further reduced to 30% and held for 15 min. The column was then reconditioned with the initial gradient for 5 min. MRM transitions were constructed for comparative analysis of various polar lipids. Quantification of individual lipid species were carried out by referencing to spiked internal standards; namely PC-14:0/14:0, PC34:1-d31, PE-14:0/14:0, PE34:1-d31, PS-14:0/14:0, PA-17:0/17:0, PG-14:0/14:0, d7-DGTS, LPE-17:1, CL-22:1(3)-14:1 which were obtained from Avanti Polar Lipids (Alabaster, AL) and LIPID MAPS. 16:0-PI was purchased from Echelon Biosciences, Inc. (Salt Lake City, UT) and used together with PI34:1-d31 (LIPID MAPS) for PI quantitation. Qualitative deuterated lipid standards from LIPID MAPS were pre-corrected based on available quantitative lipid standards prior to their use for quantitation.

For reverse phase LC/MS, glycerol lipids (MAG, DAG and TAGs) were analyzed using a modified version of reverse phase (RP)-HPLC/ESI/MS/MS as reported previously [[6](#_ENREF_6)]. In brief, separation of the aforementioned lipids was carried out on a Phenomenex Kinetex 2.6 μm-C18 column (internal diameter 4.6 × 100 mm) using an isocratic mobile phase of chloroform:methanol:0.1M ammonium acetate (100:100:4) at a flow rate of 160 μl min^-1^ for 20 min. Based on neutral loss MS/MS techniques, the levels of TAGs were calculated relative to the intensity of spiked d5-TAG 48:0 internal standard (CDN Isotopes), while MAG and DAG species were quantified using d5-MAG20:4, d6-DAG16:0/16:0, d5-DAG18:1/18:1 as an internal standard (Avanti Polar Lipids). Separation of galactolipids (MGDGs and DGDGs) were conducted using Phenomenex Kinetex 2.6μm-C18 column (internal diameter 4.6 × 100 mm) with an isocratic gradient of chloroform: methanol: 2% of 50 mM sodium acetate (49: 49: 2) at a flow rate of 160 μL min^-1^ for 25 min as reported previously [[7](#_ENREF_7)].

**cDNA cloning of *ApMYB6***

A cDNA fragment of *ApMYB6* (966 bp) was obtained by PCR using one pair of primers contained recombination sites with KOD DNA polymerase (TOYOBO): the sense primer (5'-tggacgagctgtacaagactagtATGGGAAAACCGGACGAGTACA-3') and the antisense primer (5'-cgagcggcctaagaatgcggccgcTCACAAGTCGGTCGGGGAG-3'). The PCR product was introduced into the vector constructed by our lab using Seamless Cloning Kit (Beyotime). The sequence was submitted to GenBank (Accession number: MT863608).

**References**

1. Gao C, Wang Y, Shen Y, Yan D, He X, Dai J, Wu Q. Oil accumulation mechanisms of the oleaginous microalga *Chlorella protothecoides* revealed through its genome, transcriptomes, and proteomes. BMC Genom. 2014; 15:582.

2. Tanaka T, Maeda Y, Veluchamy A, Tanaka M, Abida H, Marechal E, Bowler C, Muto M, Sunaga Y, Tanaka M *et al*. Oil accumulation by the oleaginous diatom *Fistulifera solaris* as revealed by the genome and transcriptome. Plant Cell. 2015; 27(1):162-176.

3. Welti R, Li W, Li M, Sang Y, Biesiada H, Zhou HE, Rajashekar CB, Williams TD, Wang X. Profiling membrane lipids in plant stress responses. J Biol Chem. 2002; 277(35):31994-32002.

4. Lam SM, Tong L, Duan X, Petznick A, Wenk MR, Shui G. Extensive characterization of human tear fluid collected using different techniques unravels the presence of novel lipid amphiphiles. J Lipid Res. 2014; 55(2):289-298.

5. Lam SM, Wang Z, Li J, Huang X, Shui G. Sequestration of polyunsaturated fatty acids in membrane phospholipids of *Caenorhabditis elegans* dauer larva attenuates eicosanoid biosynthesis for prolonged survival. Redox Biol. 2017; 12:967-977.

6. Shui G, Guan XL, Low CP, Chua GH, Goh JS, Yang H, Wenk MR. Toward one step analysis of cellular lipidomes using liquid chromatography coupled with mass spectrometry: application to *Saccharomyces cerevisiae* and *Schizosaccharomyces pombe* lipidomics. Mol BioSyst. 2010; 6(6):1008-1017.

7. Cheong WF, Wenk MR, Shui G. Comprehensive analysis of lipid composition in crude palm oil using multiple lipidomic approaches. J Genet Genomics. 2014; 41(5):293-304.
